# Assessment of cervical vascularization density in patients with locally advanced squamous cell cervical carcinoma evaluated in colour Doppler and power Doppler functions

**DOI:** 10.1007/s00404-021-06161-0

**Published:** 2021-09-29

**Authors:** Adam Tomalczyk, Bartłomiej Tomasik, Jacek Fijuth, Malgorzata Moszynska-Zielinska, Leszek Gottwald

**Affiliations:** 1 Department of Radiology, Copernicus Memorial Hospital, Pabianicka 62, 93-513 Lodz, Poland; 2 Department of Biostatistics and Translational Medicine, Medical University of Lodz, Lodz, Poland; 3 Department of Radiation Oncology, Dana-Farber Cancer Institute, Harvard Medical School, Boston, MA 02215 USA; 4 Department of Radiotherapy, Medical University of Lodz, ul. Paderewskiego 4, 93-509 Lodz, Poland; 5 Department of Teleradiotherapy, Regional Cancer Centre, Copernicus Memorial Hospital of Lodz, Lodz, Poland

**Keywords:** Cervical carcinoma, Locally advanced, Colour Doppler, Power Doppler, Recurrence, Survival

## Abstract

**Purpose:**

The aim of the prospective study was to assess changes during treatment and prognostic significance of cervical vascularization density in patients with cervical squamous cell carcinoma (SCC) staged II B and III B and to find relationship of cervical vascularization density with tumour diameter, grading, staging and age of patients.

**Methods:**

The study group included 50 patients who underwent transvaginal Doppler ultrasonography prior to chemoradiotherapy, after external beam radiation therapy (EBRT) and 6 weeks after HDR brachytherapy. The colour Doppler (CD) vascularity index (CDVI) and the power Doppler (PD) vascularity index (PDVI) in cervical tumour were examined.

**Results:**

CDVI and PDVI values decreased significantly during radiotherapy (0.13 (95% CI 0.09–0.16); 0.09 (95% CI 0.07–0.11) and 0.05 (95% CI 0.03–0.06) for CDVI (*p* < 0.001) and 0.26 (95% CI 0.22–0.31); 0.18 (95% CI 0.14–0.22) and 0.08 (95% CI 0.06–0.11) for PDVI (*p* < 0.001)). No statistically significant associations of CDVI and PDVI with tumour diameter, grading, staging and age of patients were found. The higher (above median) CDVI values before EBRT were associated with better OS (*p* = 0.041). The higher (above median) PDVI values before EBRT were associated with superior DFS (*p* = 0.011) and OS (*p* < 0.001). DFS and OS did not differ significantly regarding CDVI and PDVI values after EBRT and after the treatment.

**Conclusions:**

In the study group, cervical vascularization density evaluated in CD and PD functions did not depend on tumour diameter, grading, staging and age of patients and decreased during radiotherapy. The prognosis for our patients with CDVI and PDVI before the treatment above the median value was better than compared to patients with these parameters below the median value.

## Introduction

In locally advanced cervical squamous cell carcinoma (SCC), the treatment of choice is chemoradiotherapy, and the prognosis depends on a number of factors related to the biology of the tumour and the general condition of patients [1, 2]. Despite continuous improvement of radiotherapy techniques and progress in systemic treatment, the results of treatment in these patients are still unsatisfactory. Staging, histology and grading are parameters which characterise tumour evaluated in clinical practice [2, 3]. Other parameters which will help to identify patients with better and worse prognosis are being searched [3, 4].

Tumour angiogenesis is the production of new tumour vessels. It has gained much attention in oncology in recent years. Some studies have demonstrated that it is an essential process for tumour growth, correlated with tumour metastatic potential and can be considered as an independent prognostic factor in cervical carcinoma [5–8]. The assessment of tumour vascularity could, therefore, become a novel means of predicting tumour aggressiveness.

Transvaginal ultrasonography is a non-invasive method that allows accurate study of the pelvic organs [9]. The addition of colour and power Doppler has further enhanced the diagnostic potential of ultrasonography [10]. Transvaginal Doppler ultrasonography allows an in vivo non-invasive and prospective assessment of tumour vascularization. Colour Doppler (CD) has demonstrated that increased angiogenesis plays an important role in the tumourigenesis cervical malignancies [11, 12]. Power Doppler (PD) is a useful tumour flow mapping technique with several advantages, including relative angle and velocity independence, extended dynamic range and higher sensitivity [13]. However, the clinical value of changes of CD and PD parameters during treatment in cervical tumour of patients with locally advanced cervical SCC are not clear.

## Objectives

The aim of the prospective study was to assess changes during treatment and prognostic significance of cervical vascularization density in patients with cervical squamous cell carcinoma (SCC) staged II B and III B and to find relationship of cervical vascularization density with tumour diameter, grading, staging and age of patients.

### Materials and methods

The study group consisted of 50 patients with cervical SCC staged II B and III B treated at the Department of Teleradiotherapy of the Copernicus Memorial Hospital of Lodz between 2015 and 2017. All patients underwent computed tomography of the abdomen and magnetic resonance imaging of the pelvis prior to the treatment to evaluate the staging. Only patients with negative iliac and paraaortic adenopathy in radiologic imaging were included into the study. In this selected group of patients, operative staging was not performed. Detailed data of the studied population are presented in Table 1.

**Table 1:** Characteristics of the study group

Selected clinical and pathological data	*n*	%
Age (years)		
≤ 50	14	28%
51–65	22	44%
> 65	14	28%
Pregnancies		
No	5	10%
Yes	45	90%
Deliveries		
No	7	14%
Yes	43	86%
Tumour diameter		
< 4 cm	22	44%
≥ 4 cm	28	56%
Tumour volume		
< 20 cm^3^	23	46
20–40 cm^3^	18	36
> 40 cm^3^	9	18
WHO grading		
G 1 + G 2	39	78%
G 3	11	22%
FIGO staging		
IIB	37	74%
IIIB	13	26%
Treatment		
EBRT (60 Gy/2 Gy)	1	2%
EBRT (44 Gy/2 Gy) + HDR BT	3	6%
EBRT (44 Gy/2 Gy) + P + HDR BT	46	92%
Relapse		
None	37	74%
Only local	3	6%
Only distant	7	14%
Survival		
Local and distant	3	6%
Alive	40	80%
Dead	10	20%
Total	50	100

The treatment scheme involved application of external beam radiotherapy (EBRT) to primary tumour mass, the entire uterus, adequate vagina, parametrium, common iliac, internal iliac, external iliac, presacral and obturator lymph nodes of a dose up to 44 Gy, fractionated at 2 Gy, with weekly injections of cisplatin (P) at a dose of 40 mg/m^2^. In patients with renal failure and elevated serum creatinine concentration, only EBRT was applied. After EBRT with or without P was completed, high-dose rate brachytherapy (HDR BT) was implemented, fractionated at one application of 7 Gy weekly for 4 weeks up to a total dose of 28 Gy, or EBRT was continued to the uterus and primary infiltrating tissues, up to a total dose of 60 Gy. Follow-up monitoring was carried out in an oncological outpatient clinic until December 31, 2018.

All patients underwent transvaginal Doppler ultrasonography (TV DU). We predetermined TV DU in three time points. First examination was conducted prior to the treatment and to assess initial Doppler parameters of the tumour. Second examination was performed before HDR BT to assess the changes in cervical vascularization density resulted due to EBRT. Third examination was made during first follow-up visit 6 weeks after the treatment was finished and it showed cervical vascularization density after radiotherapy.

**Fig. 1:**
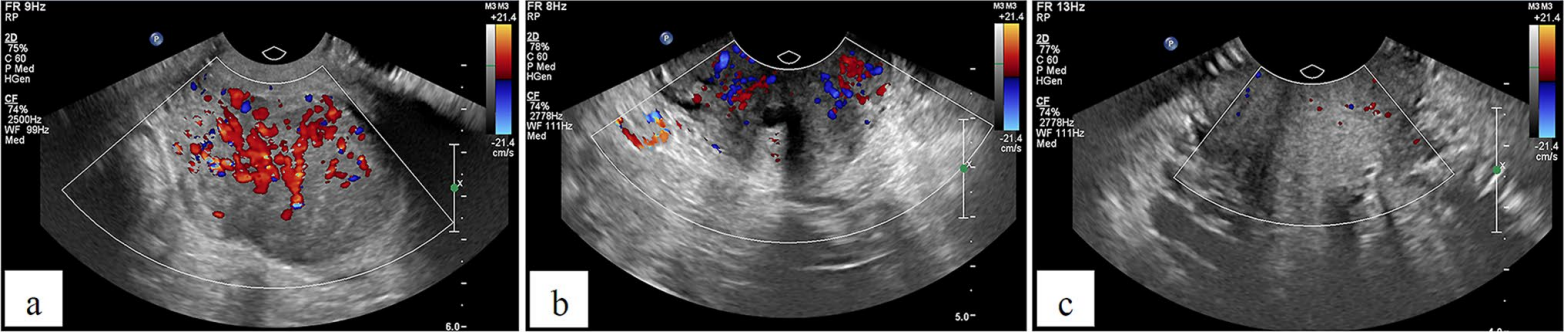
CDVI in the patient with locally advanced cervical SCC: prior to the treatment (**a**), before HDR BT (**b**), after the treatment (**c**)

**Fig. 2:**
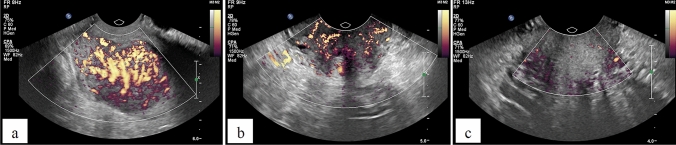
PDVI in the patient with locally advanced cervical SCC: prior to the treatment (**a**), before HDR BT (**b**), after the treatment (**c**)

The TV US examinations were performed by the same examiner using Philips iU22 with 10 MHz endovaginal probe C10-3v. The scanning protocol was the same throughout the study period and all examinations began with the same setting of the ultrasound system. In TV US, the tumour parameters were estimated; afterwards, a Doppler frame was put in and precisely adjusted to comprise the whole tumour. To assess vascularity in CD CF (Colour Flow), 74% settings for frequency of 2500 Hz were used. To assess vascularity in PD CPA (Colour Power Angio), 70% settings for frequency of 1500 Hz were used. These settings enabled to assess tumour vessels colouring undisrupted by background noise. The imaging registration of these two techniques was carried out at the top of vasoconstriction, by the biggest flows. Afterwards, the ratio of colour pixels/all pixels of the examined area from the cervical tumour, defined as the CD vascularity index (CDVI; Fig. 1) and PD vascularity index (PDVI; Fig. 2), were calculated.

The statistical analysis was carried out using Statistica 13.1 software (Statsoft, Tulsa, OK, US). Nominal variables were expressed as percentages and analysed using the Chi-square test with appropriate corrections (the Yates's correction for continuity or the Fisher exact test), if needed. The normality of the distribution of continuous variables was verified with the Shapiro–Wilk test. Depending on the data distribution, continuous variables were compared using *t* test or Mann–Whitney *U* test. Paired comparisons across three timepoints were analysed using the repeated-measures analysis of variance and data were represented as mean together with 95% confidence interval (95% CI). Sphericity assumption was tested using the Mauchly’s test. A *p* value < 0.05 was considered statistically significant. The group size was estimated using standard power analysis methods. For the purpose of outcome analyses, disease-free survival (DFS) was defined as the period of time between the day of implementation of chemoradiotherapy and the day of the second cancer, locoregional recurrence, distant metastases or death from any cause. Overall survival (OS) was defined as the period of time between the commencement of chemoradiotherapy and the day of death from any cause. Estimated DFS and OS were presented using Kaplan–Meier survival curves compared using the log-rank test. We assumed that clinically relevant differences would need to exceed 1/3 standard deviation of the analysed colour Doppler and power Doppler parameters. Therefore, to maintain statistical power > 80% with a predetermined type 1 error probability < 0.05 we calculated that a group of 45 patients is the required minimum. To take the missing data into account, this group was increased by 10%, which resulted in inclusion of five additional patients.

The study was approved by the Bioethics Commission of the Medical University of Lodz No. RNN/94/15/KE.

## Results

**Fig. 3:**
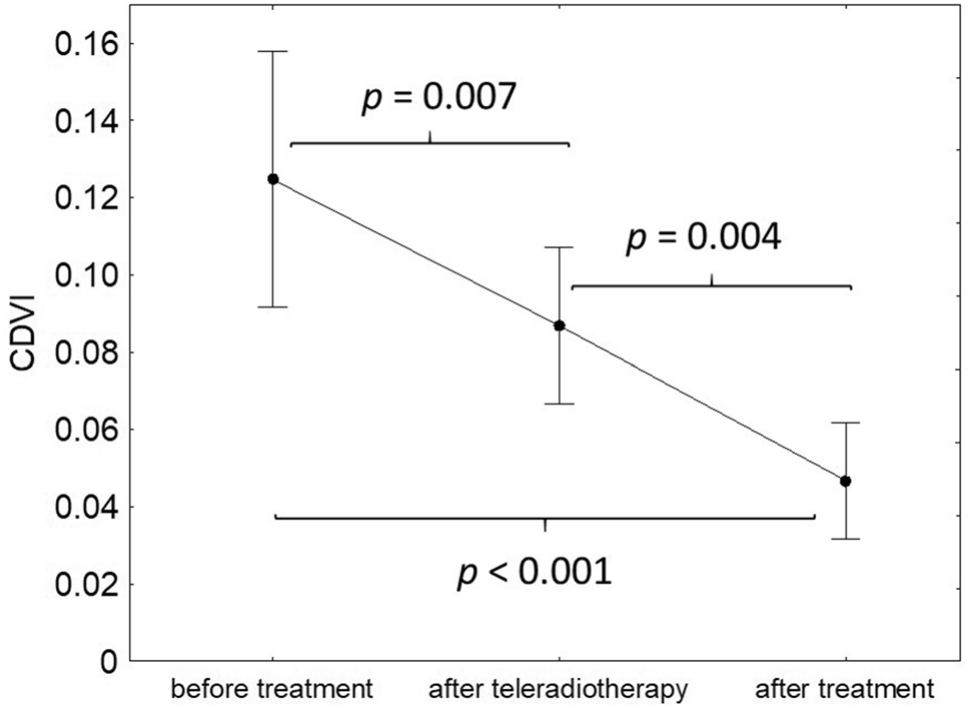
Changes of CDVI during the treatment. Black dot represents mean and whiskers represent 95% confidence intervals

In the study group, CDVI values decreased during radiotherapy (*p* < 0.001; Fig. 3). The CDVI value before the onset of the treatment was significantly higher than compared to its value after EBRT, as well as 6 weeks after the treatment (0.13 (95% CI 0.09–0.16) vs 0.09 (95% CI 0.07–0.11), *p* = 0.007 and 0.13 (95% CI 0.09–0.16) vs 0.05 (95% CI 0.03–0.06), *p* < 0.001, respectively). The CDVI value in the second examination was significantly higher than compared to its value in the third examination (0.09 (95% CI 0.07–0.11) vs 0.05 (95% CI 0.03–0.06), *p* = 0.004). Successive examinations did not confirm any statistically significant associations of CDVI decrease during treatment and initial tumour diameter (*p* = 0.309), grading (*p* = 0.486), staging (*p* = 0.946) and the age of patients (*p* = 0.155).

**Fig. 4:**
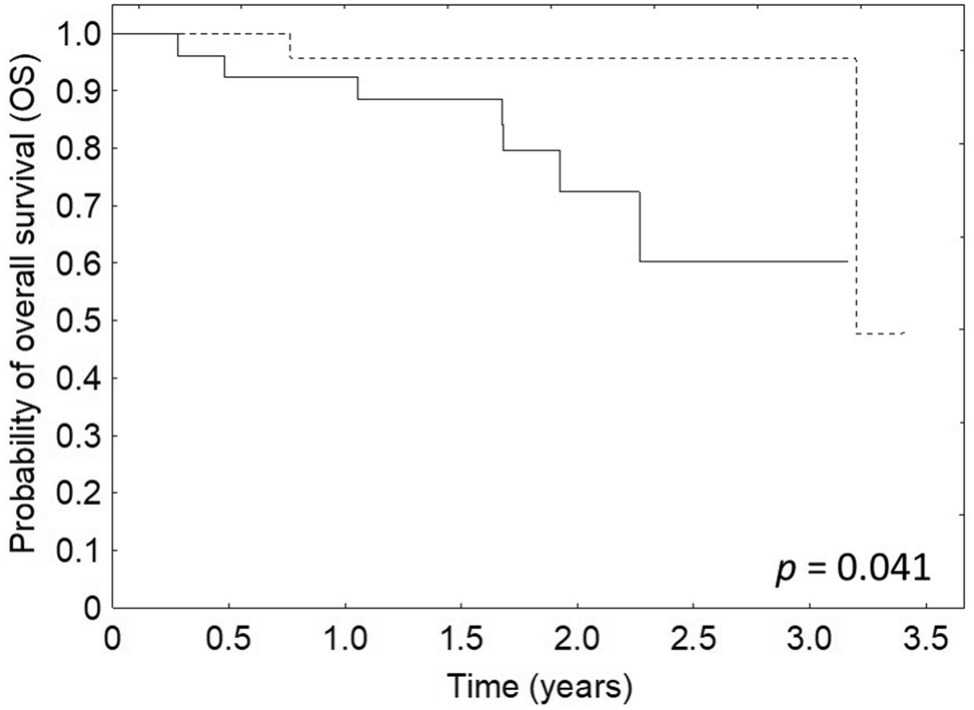
Relationship between OS and CDVI before the treatment (solid line—patients with CDVI below the median value, dotted line—patients with CDVI above the median value)

The CDVI values before EBRT were not connected to DFS (*p* = 0.291), but were associated with better OS (*p* = 0.041). OS of patients with pre-treatment CDVI values above the median value (0.1) was better than compared to patients with CDVI below the median value (Fig. 4). DFS and OS were not connected to CDVI values after EBRT (*p* = 0.139 and *p* = 0.125, respectively) and after the treatment (*p* = 0.997 and *p* = 0.252, respectively).

**Fig. 5:**
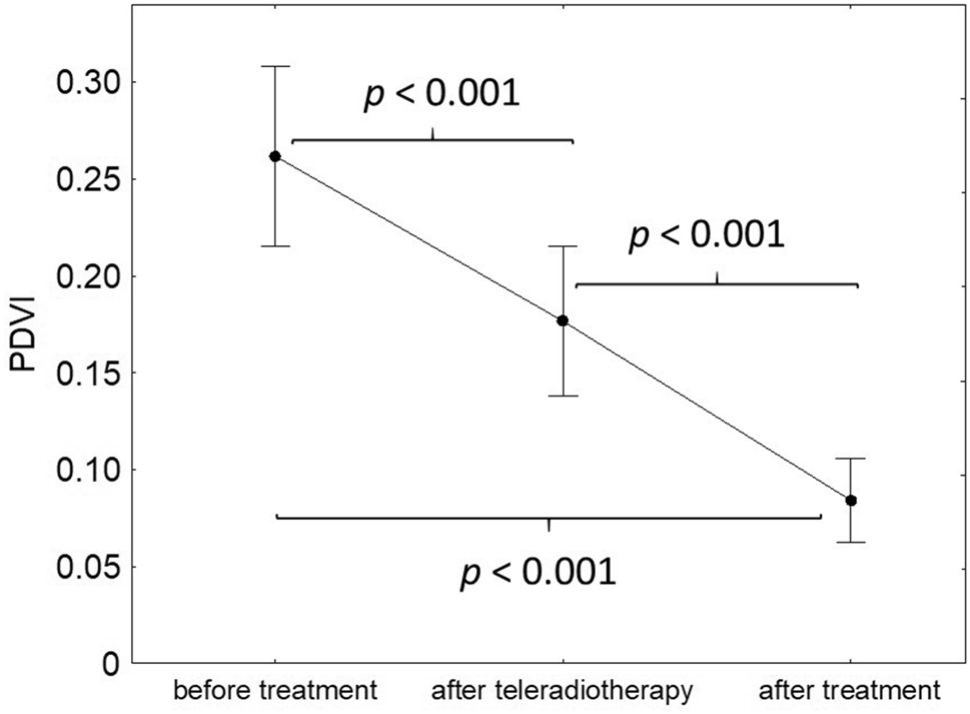
Changes of PDVI during the treatment. Black dot represents mean and whiskers represent 95% confidence intervals

Similar to CDVI, PDVI values decreased during radiotherapy (*p* < 0.001; Fig. 5) in patients with locally advanced cervical SCC. The PDVI value before the onset of the treatment was significantly higher than compared to its value after EBRT, as well as 6 weeks after the treatment (0.26 (95% CI 0.22–0.31) vs 0.18 (95% CI 0.14–0.22), *p* < 0.001 and 0.26 (95% CI 0.22–0.31) vs 0.08 (95% CI 0.06–0.11), *p* < 0.001, respectively). The PDVI value in the second examination was significantly higher than compared to its value in the third examination (0.18 (95% CI 0.14–0.22) vs 0.08 (95% CI 0.06–0.11), *p* < 0.001). In successive examinations, no statistically significant associations of PDVI decrease during treatment and initial tumour diameter (*p* = 0.741), grading (*p* = 0.736), staging (*p* = 0.688) and the age of patients (*p* = 0.310) were found.

**Fig. 6:**
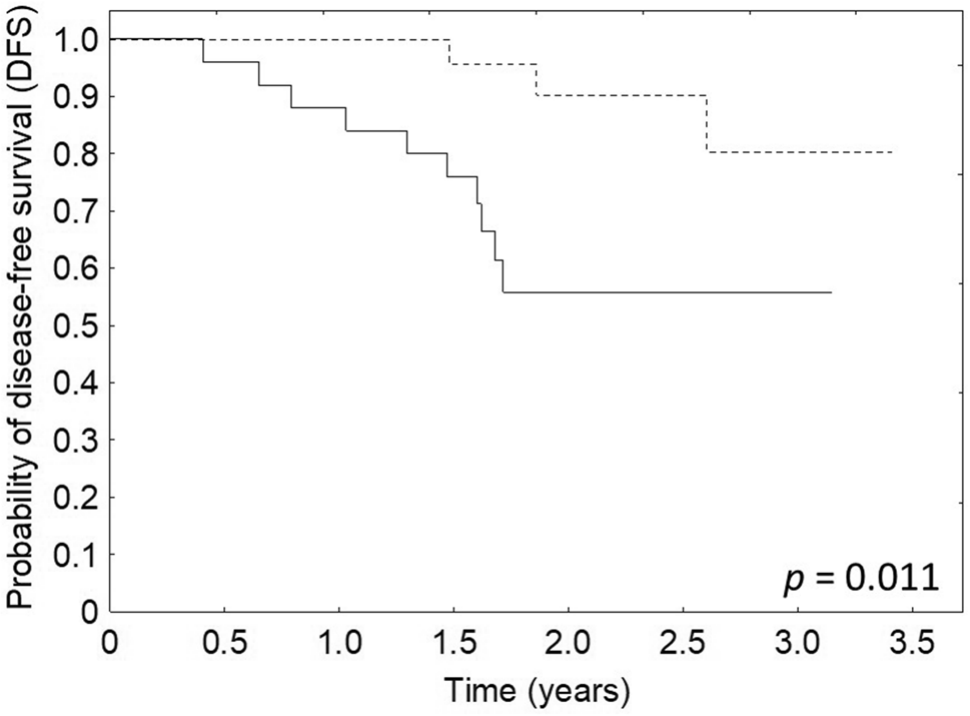
Relationship between DFS and PDVI before the treatment (solid line—patients with CDVI below the median value, dotted line—patients with PDVI above the median value)

**Fig. 7:**
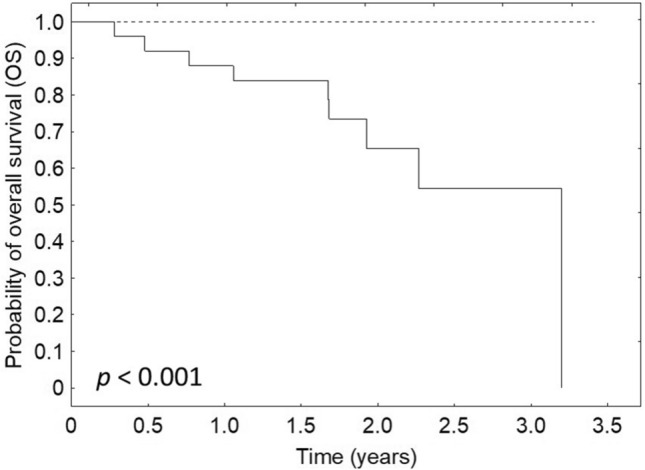
Relationship between OS and PDVI before the treatment (solid line—patients with CDVI below the median value, dotted line—patients with CDVI above the median value)

The PDVI values before EBRT were associated with DFS and OS (*p* = 0.011 and *p* < 0.001, respectively). Both DFS and OS of patients with pre-treatment PDVI values above the median value (0.24) were better than compared to patients with PDVI below the median value (Figs. 6, 7). DFS and OS were not connected to PDVI values after EBRT (*p* = 0.149 and *p* = 0.453, respectively) and after the treatment (*p* = 0.743 and *p* = 0.756, respectively).

## Discussion

Although many authors examined ultrasonographic Doppler parameters in tumours of cervical patients, our study is valuable, because it was prospective, performed by the same examiner, all the ultrasonographic examinations were made at predetermined time points related to the treatment and all the patients were treated according to the same protocol by one medical team.

In our study, tumour vascularization did not depend on the tumour diameter, histological grading, staging and the age of the patients, but literature data are ambiguous. Similar to our results, Testa et al. in 77 patients with cervical SCC did not find significant correlations between 3D colour power angiography parameters and clinicopathological characteristics of cervical tumour or between 3D vascular parameters and biological factors [7]. Belitsos et al. examined 71 patients with cervical carcinoma and observed tumour vascularization being not significantly different in relation to grade, histology, presence of positive lymph nodes or lymphovascular space involvement. However, vascularization index was significantly higher in patients with stages IIIB–IV than in patients with less advanced disease [14]. Some authors presented different observations. Alcázar et al. examined 56 patients with cervical carcinoma and observed higher tumour vascularization in poorly differentiated, advanced cervical carcinomas with large primary tumours [15]. Similarly, Jurado et al. among 27 patients with early-stage cervical carcinoma found before surgery significant association between tumour abundant vascularization and depth of stromal invasion > 10 mm, tumour diameter > 17.5 mm and parametrial involvement [16]. Additionally, Jurado et al. [16] described significant association between cervical tumour vascularization, pelvic lymph node metastases and lymphovascular space involvement, while in patients treated by Alcázar et al. [15], this relationship was not proven.

The conducted analysis showed that in our study group, poorly vascularized cervical carcinomas had a worse response to treatment than well-vascularized tumours. The vascular density of cervical carcinomas, assessed in CD and PD before the treatment, was associated with prognosis in patients. In both cases, their value below the median was associated with significantly shorter OS and for PDVI, additionally with significantly shorter DFS. These results confirm one of the basic principle of cancer radiobiology, saying that better tumours vascularized are three times more radiosensitive than hypoxic neoplasms. Therefore, hypoxia is regarded as a factor of unfavorable prognosis. The direct correlation between high levels of hypoxia and tumour aggressiveness has been shown in numerous studies. Patients with hypoxic tumours need a higher total dose of radiotherapy to recover [17, 18].

The variability of vascularization in cervical carcinomas during treatment was the topic of interest in many studies. We observed, that both CDVI and PDVI decreased significantly at subsequent time points during the treatment. Similarly, Pirhonen et al. in patients with cervical carcinoma staged IIA–IVB treated with EBRT described significant decrease in tumour vascularization [19]. These results were confirmed by other authors [20–22].

Literature data show that better prognosis is connected with patients with decreased tumour vascularity during oncological treatment rather than with persistent vascularization density. Chen el al. examined 25 patients with bulky early-stage cervical carcinoma treated with neoadjuvant chemotherapy followed by surgery. After chemotherapy higher PDVI values were noted in non-responders and lower PDVI values were observed in responders [20]. Similarly, Xu et al. performed serial three-dimensional PD examinations in 32 patients with advanced-stage cervical carcinoma treated with chemoradiotherapy. During the treatment in patients classified as complete responders PDVI decreased, but no significant differences in PDVI values were seen in the group of partial responders [22]. Similar results reported Tesla et al. among 88 patients with cervical carcinoma of any histology staged IB2-IVA treated with chemoradiotherapy [10]. Pirhonen et al. presented compatible results after radiotherapy, the decrease of tumour vascularization during radiotherapy was associated with better outcome, whereas persistence of excessive vascularity at the end of radiation was associated with modest therapeutic response [19]. Additionally, Huang et al. in 37 patients with cervical carcinoma staged IB1–IIB who were undergoing radiotherapy found cervical intratumoral vascularization being disappeared within 3 months following radiotherapy, except in one patient with persistent disease [21]. Generally, it is postulated, that in cases with total response to the treatment a significant decrease in tumour vascularity occur, and in cases with only partial regression or no regression of cervical carcinoma tumour vascularity parameters remain unchanged.

In our study of all the assessed Doppler parameters, only cervical vascularization density before the treatment, but not after EBRT and 6 weeks after the treatment, was associated with prognosis in patients with locally advanced cervical carcinoma. According to the prognostic significance of single Doppler examination before treatment of cervical tumour vascularity in patients with locally advanced cervical carcinoma the literature data are ambiguous. Qin et al. conducted a study on a group of 61 patients with locally advanced cervical carcinoma who underwent neoadjuvant chemotherapy, followed by surgery or radiation. They revealed that before the initial chemotherapy, the vascularization index, flow index and vascularization flow index were significantly higher in clinical responders than in non-responders [23]. Interestingly, Alcazar et al. noted that in 49 patients with advanced-stage cervical carcinoma a single 3D-PD sonographic assessment of tumour size and vascularization before the treatment seems to have a limited value for predicting tumour response to chemoradiation therapy and for predicting recurrence [24].

Our results cannot be generalized to the whole population because the limitation of our study is small study group. No control group and only univariate analyses additionally limited the importance of our results. Further prospective studies in larger population of patients with locally advanced squamous cell cervical carcinoma are needed to precisely evaluate the value of cervical vascularization density in these patients.

## Conclusions

1. In the study group, cervical vascularization density evaluated in CD and PD functions, did not depend on tumour diameter, grading, staging and age of patients and decreased during radiotherapy.
2. The prognosis for our patients with CDVI and PDVI before the treatment above the median value was better than compared to patients with these parameters below the median value.

## Data Availability

Yes.

## Code availability

Yes.
